# AI-driven convolutional neural networks for accurate identification of yellow fever vectors

**DOI:** 10.1186/s13071-024-06406-2

**Published:** 2024-08-02

**Authors:** Taís Oliveira de Araújo, Vinicius Lima de Miranda, Rodrigo Gurgel-Gonçalves

**Affiliations:** 1 Programa de Pós-Graduação em Medicina Tropical, Faculdade de Medicina, Universidade de Brasília, Brasilia, DF Brasil; 2 Laboratório de Parasitologia Médica e Biologia de Vetores, Faculdade de Medicina, Universidade de Brasília, Brasilia, DF Brasil

**Keywords:** Deep learning, Machine learning, Culicidae

## Abstract

**Background:**

Identifying mosquito vectors is crucial for controlling diseases. Automated identification studies using the convolutional neural network (CNN) have been conducted for some urban mosquito vectors but not yet for sylvatic mosquito vectors that transmit the yellow fever. We evaluated the ability of the AlexNet CNN to identify four mosquito species: *Aedes serratus*, *Aedes scapularis*, *Haemagogus leucocelaenus* and *Sabethes albiprivus* and whether there is variation in AlexNet’s ability to classify mosquitoes based on pictures of four different body regions.

**Methods:**

The specimens were photographed using a cell phone connected to a stereoscope. Photographs were taken of the full-body, pronotum and lateral view of the thorax, which were pre-processed to train the AlexNet algorithm. The evaluation was based on the confusion matrix, the accuracy (ten pseudo-replicates) and the confidence interval for each experiment.

**Results:**

Our study found that the AlexNet can accurately identify mosquito pictures of the genus *Aedes*, *Sabethes* and *Haemagogus* with over 90% accuracy. Furthermore, the algorithm performance did not change according to the body regions submitted. It is worth noting that the state of preservation of the mosquitoes, which were often damaged, may have affected the network’s ability to differentiate between these species and thus accuracy rates could have been even higher.

**Conclusions:**

Our results support the idea of applying CNNs for artificial intelligence (AI)-driven identification of mosquito vectors of tropical diseases. This approach can potentially be used in the surveillance of yellow fever vectors by health services and the population as well.

**Graphical abstract:**

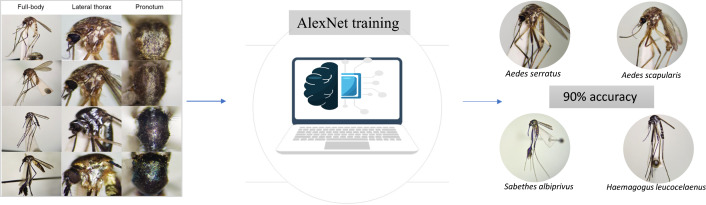

**Supplementary Information:**

The online version contains supplementary material available at 10.1186/s13071-024-06406-2.

## Background

Mosquito-borne diseases are a major public health concern. More than half of the world’s population is exposed to arboviruses such as yellow fever, dengue and Zika. Currently, 47 countries, including 34 in Africa and 13 in Central and South America, are endemic or have endemic regions for yellow fever [1, 2]. These pathogens are mainly transmitted by mosquito bites, with *Aedes aegypti* and *Aedes albopictus* species being the most important urban vectors of these arboviruses [3, 4]. Sylvatic mosquito species from the *Sabethes* and *Haemagogus* genera have been found to be naturally infected with the yellow fever virus and other arboviruses, for which no specific therapeutic agents exist [5–11].

One approach to prevent the spread of these diseases is by controlling the spread of the virus’ vector. Traditional mosquito surveillance relies on catches and species identification, which require regular manual inspection and dedicated personnel, making large-scale monitoring difficult and expensive. Additionally, identifying mosquitoes is a difficult task that demands specialized knowledge due to the vast range of morphological characteristics found in the Culicidae family, which includes all mosquitoes [12]. New approaches that rely on smartphones and the internet can enable new community and digital observatories with the task of species identification. These observatories allow individuals to submit photos of mosquitoes they come across. In addition, smartphone cameras are improving macro photography, which can produce high-quality images of small organisms such as mosquitoes. However, manually inspecting each picture is not a feasible long-term solution due to the large volume of pictures and professionals needed for the task [13–15].

Deep learning methods based on a convolutional neural networks (CNNs) have shown promise for mosquito identification [13, 14, 16–19]. CNNs simulate the human learning process for classifying pictures and extract important features from data automated without the need of human supervision [20]. AlexNet is a CNN that was pre-trained on 1.2 million images of objects, animals and plants available in the ImageNet database [21]. AlexNet has been successfully used for the identification of insect vectors [22]. Automated identification studies have been conducted for some urban mosquito vectors [13, 14, 16–19] but not yet for sylvatic mosquito vectors that transmit the yellow fever and other arboviruses [6, 11].

Therefore, considering the lack of specialised tools for the automated identification of wild yellow fever vectors using image processing, the aim of this study is to test AlexNet’s ability to identify the species *Aedes serratus*, *Aedes scapularis*, *Haemagogus leucocelaenus* and *Sabethes albiprivus*. These four mosquito species were chosen because they are confirmed vectors of the yellow fever virus (*Hg. leucocelaenus* and *Sa. albiprivus*) or because they are suspected vectors of the virus (*Ae. serratus* and *Ae. scapularis*). *Hg. leucocelaenus* was identified as a primary vector in major yellow fever outbreaks in Brazil, exhibiting a broad geographical distribution within the country. The species is found in humid and well-preserved forests and is tolerant in anthropogenic environments, which increases the human exposure to infected mosquitoes [8, 10, 23]. *Sa. albiprivus* is widely distributed in South American forests and has competence in transmitting yellow fever virus being the most important species in Argentina [11]. *Ae. serratus* and *Ae. scapularis* have been identified as potential vectors of the yellow fever, as they have been found naturally infected with the virus. However, their vectorial competence and role in the sylvatic cycle of the disease remain poorly understood [5, 6].We also asked whether there is variation in AlexNet’s ability to classify mosquitoes based on images of three different body regions. Our findings indicate that the AlexNet network can accurately identify yellow fever vectors with over 90% accuracy for the four body regions analysed, presenting great potential for the development of an application to facilitate surveillance of these vectors.

## Methods

### Picture database

To build the picture database, four mosquito species were selected available at the Laboratory of Medical Parasitology and Vector Biology at the University of Brasilia. Among them, *Sa. albiprivus* (*n* = 100) and *Hg. leucocelaenus* (*n* = 98) were vectors of the wild yellow fever virus, while *Ae. serratus* (*n* = 100) and *Ae. scapularis* (*n* = 100) were vectors of other arboviruses. A total of 565 full-body pictures were captured, including 294 of the lateral thorax and 484 of the pronotum, resulting in 1343 pictures. The Culicidae specimens were mounted on a cardboard triangle using a pin. The mosquitoes were attached to the triangle by the thoracic pleura, leaving the legs facing the pin and the upper pleura free for observation.

**Fig. 1:**
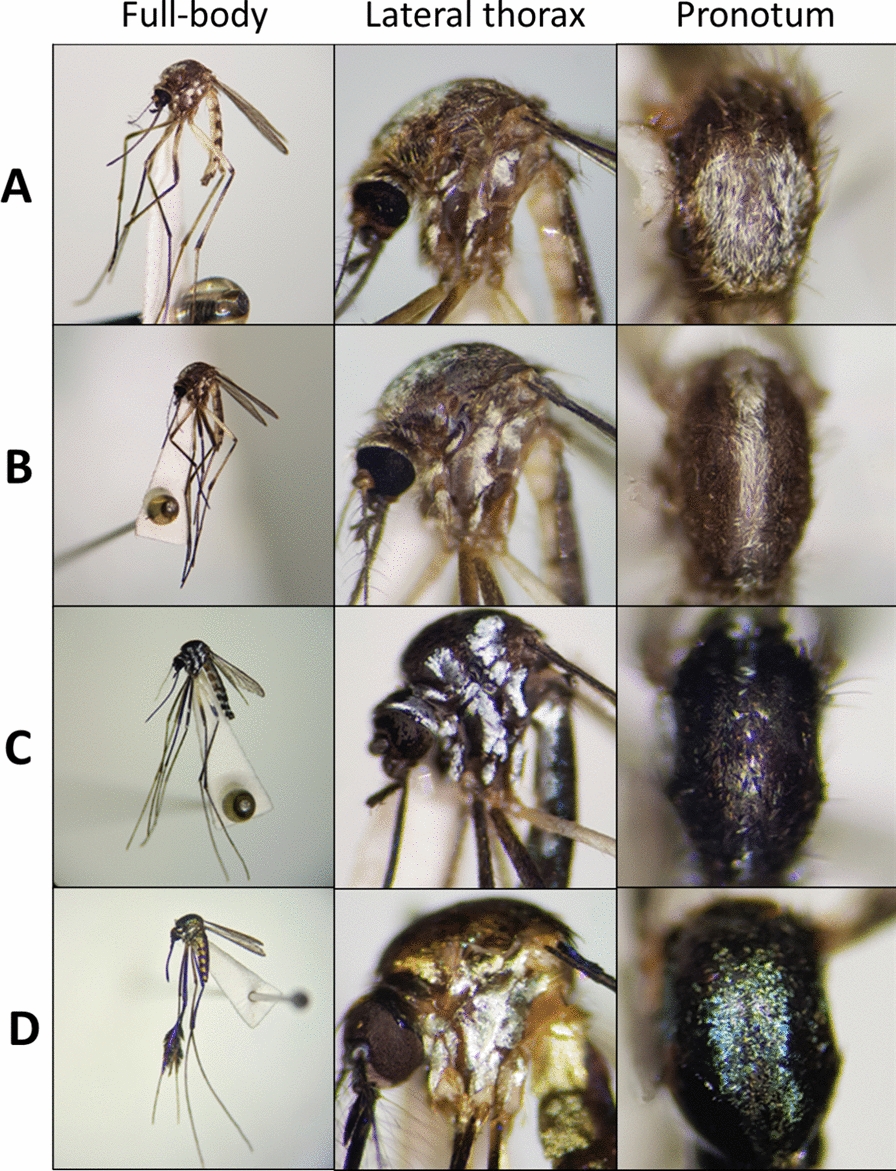
Examples of pictures used to train the AlexNet network. Mosquitoes photographed with a stereomicroscope and cell phone: *Ae. scapularis* (**A**), *Ae. serratus* (**B**),* Hg. leucocelaenus* (**C**) and *Sa. albiprivus* (**D**)

Pictures were captured using a cell phone camera (Samsung Galaxy S8, model SM-G950F, 12 mp, sensor 1/2.5, aperture size f/1.7) attached to a stereoscope (Zeiss Stemi 508, 1–10× magnification). Photographs of the full-body, pronotum and lateral view of the thorax were taken (Fig. 1), cropped in a square format (proportion 1:1) in the computer software Photoscape, selecting only the mosquito, and subsequently resized to 227 × 227 pixels using the software computational MATLAB. They were then organized into folders based on the species and experiment to be conducted.

### Algorithm

The AlexNet architecture, comprising five convolutional layers and three fully connected layers, has 60 million parameters. This network utilises 227 × 227 pixels RGB images as input. The model was pre-trained with 1.2 million images of varying resolutions, representing over 1000 classes from the ImageNet database [21]. The AlexNet architecture employs convolutional layers to extract features from images, including edges, textures and shapes. The maximum pooling layers reduce the dimensionality of the image while retaining important features. The rectified linear unit (ReLU) function introduces non-linearity to the network, enabling it to learn more complex features. Local response normalisation normalises the outputs of the convolutional layers, improving generalisation. The final classification is performed by three fully connected layers. The dropout technique, which randomly deactivates neurons during training, prevents overfitting. The Softmax function converts the network outputs into probabilities, selecting the highest probability as the final prediction [21].

### Algorithm training and testing

The AlexNet algorithm, which has already been used to identify insect vectors [16, 22], was implemented in MATLAB. The image database was divided into three sections for the experiments: 80% for training, 10% for internal validation and 10% for testing (Additional file 2: Dataset S1). The algorithm randomly selected the images for each stage. In the algorithm settings, the maximum number of epochs was set to 50 following preliminary testing. Stochastic gradient descent with a moment optimiser was employed, with default settings aside from the initial learning rate, which was set to 0.001. Four experiments were conducted with four classes in ten pseudo-replicates each (ten repetitions of training and testing using the same set of pictures). The first experiment utilised all images, including full-body, pronotum and lateral thorax. The other experiments employed separate images of each body part.

### Data analysis

The network’s performance was evaluated based on observations from confusion matrices/heat maps, the mean and confidence interval of the general accuracy (probability of correctly identifying the species independently) and the specific accuracy (probability of correctly identifying each species specific) that can be interpreted as sensitivity, depending on the context of the analysis of the algorithm for each experiment. To achieve this, we employed the ‘Hmisc’ package within the computational software R, version 4.7.1, in conjunction with the RStudio interface, version 2023.03.1.446 [24–26]. We use the following equations to calculate AlexNet performance metrics:

$$\text{General accuracy }=\frac{TP + TN}{TP + FP + TN + FN}$$

$$\text{Specific accuracy }=\frac{TP}{TP + FN}$$

TP, true positives;

TN, true negatives;

FP, false positives;

FN, false negatives.

## Results

### Confusion matrices

**Fig. 2:**
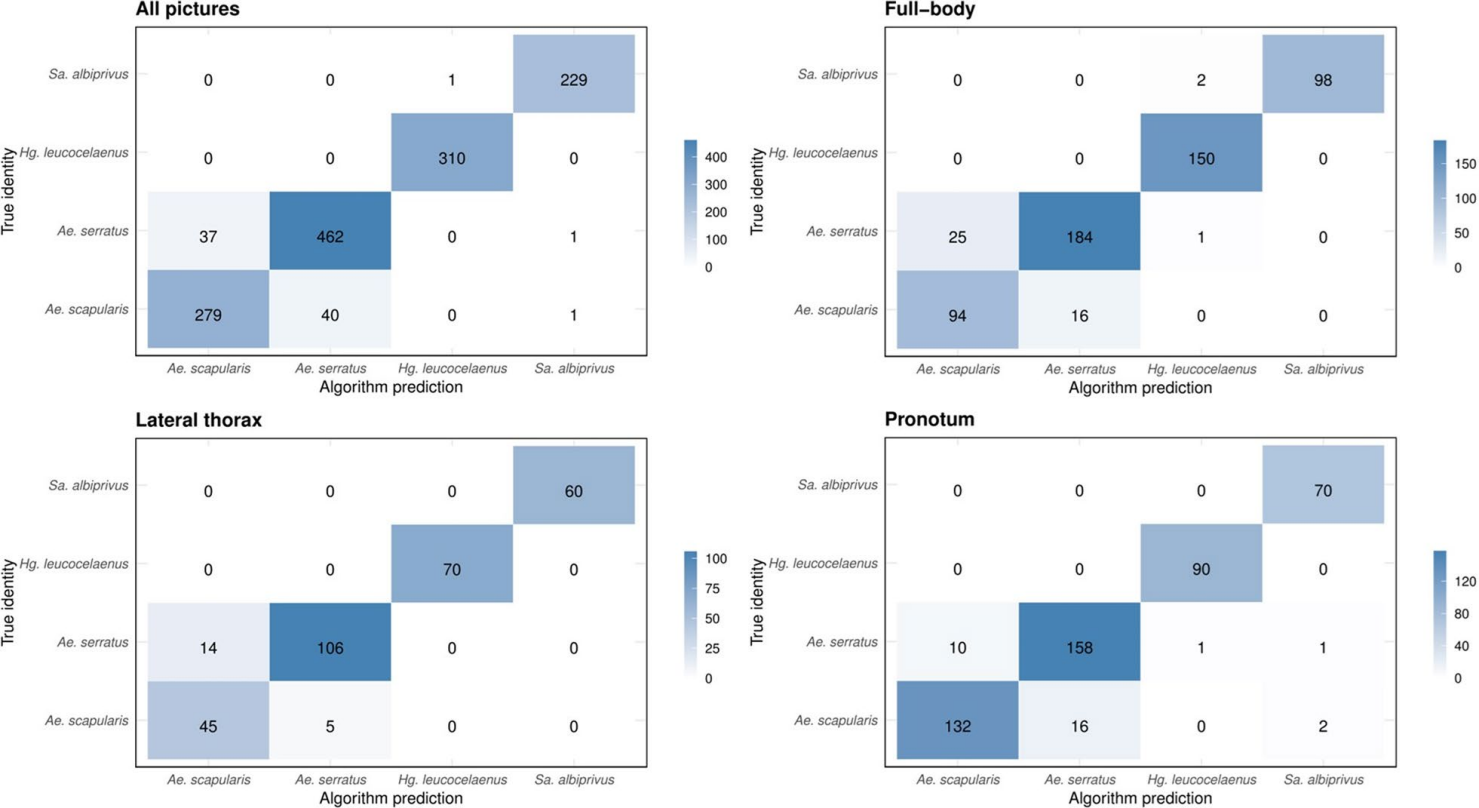
Confusion matrices showing the classification hits and misses of the 10% of the test images for the four experiments. Each run was pseudo-replicated 10×. The blue scale indicates the number of correct predictions

To gain an understanding of the performance of the AlexNet algorithm, Fig. 2 presents a series of confusion matrices, which illustrate the patterns of correct and incorrect identification of mosquito images across the ten pseudo-replicates.

### General accuracy of the algorithm

**Fig. 3:**
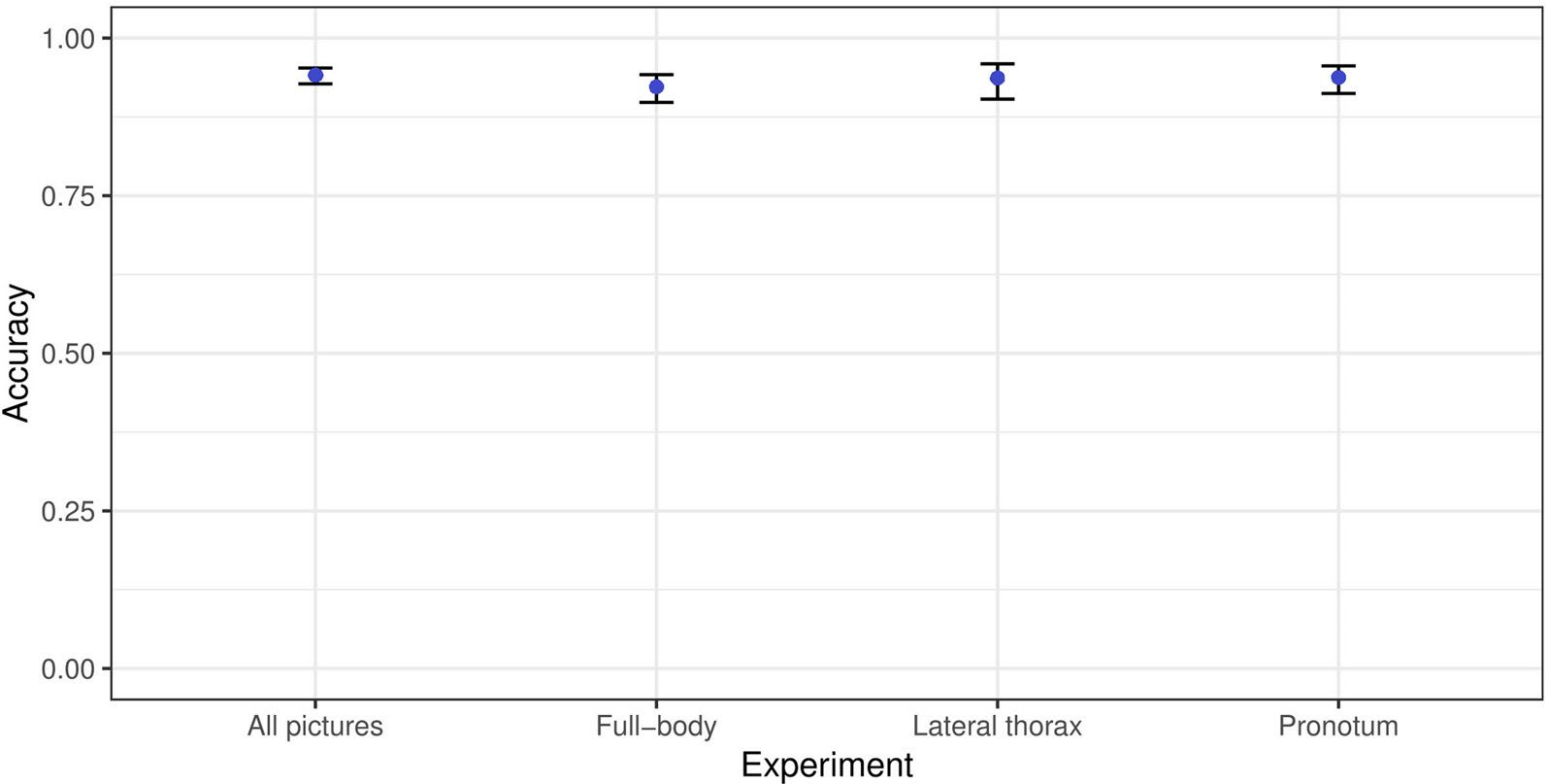
Average accuracy of the AlexNet algorithm in identifying mosquito vectors of yellow fever and other arboviruses. The experiments took into account identification from images of isolated body parts regions, with 95% confidence intervals. Each run was pseudo-replicated 10×

The performance of the algorithm was evaluated by processing test pictures after training the AlexNet algorithm. Four experiments were conducted, in which each group (full-body, lateral thorax and pronotum) was submitted to the AlexNet algorithm. Additionally, all 1343 photos corresponding to the three groups were submitted together. The average confidence interval for general accuracy was then calculated. The AlexNet algorithm demonstrated the highest general accuracy in identifying mosquitoes when all pictures were analysed together, providing the algorithm with the most comprehensive information about the mosquito’s body. Each run was repeated ten times, achieving an average general accuracy of 94% (95% confidence interval (CI) 90–97). The run with the total body group achieved an average general accuracy of 92.3% (95% CI 83–97), while with the lateral view group the value was 93.7% (95% CI 79–98). Finally, with the pronotum group, the average general accuracy was 93.8% (95% CI 83–98). The smallest confidence intervals were observed with pictures of all parts of the body (Fig. 3; see Additional file 1: Table S1).

### Algorithm-specific accuracy

**Fig. 4:**
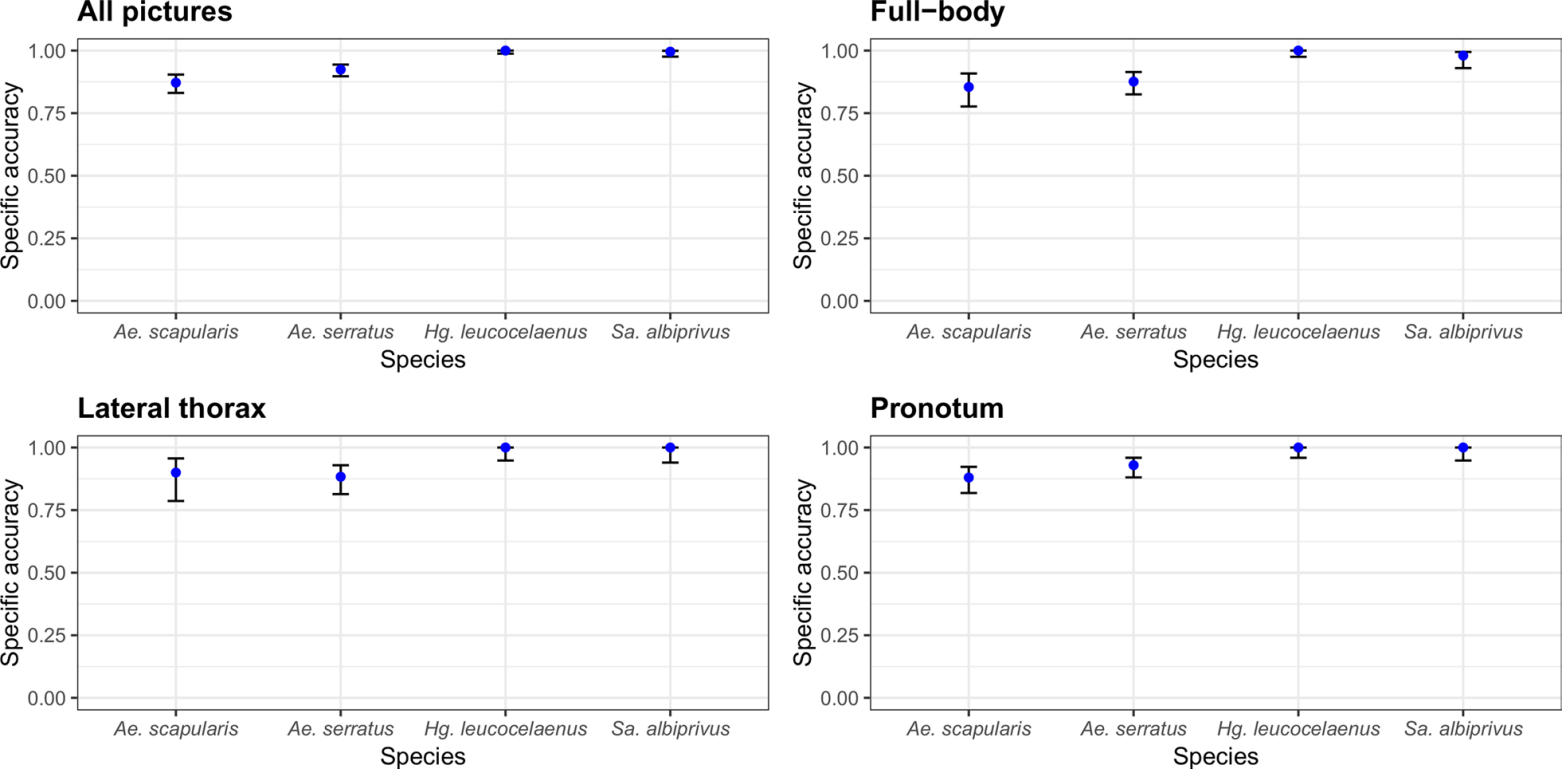
Average accuracy of the AlexNet algorithm in interspecies identification. The experiments took into account identification from images of isolated body parts regions, with 95% confidence intervals. Each run was pseudo-replicated 10×

**Fig. 5:**
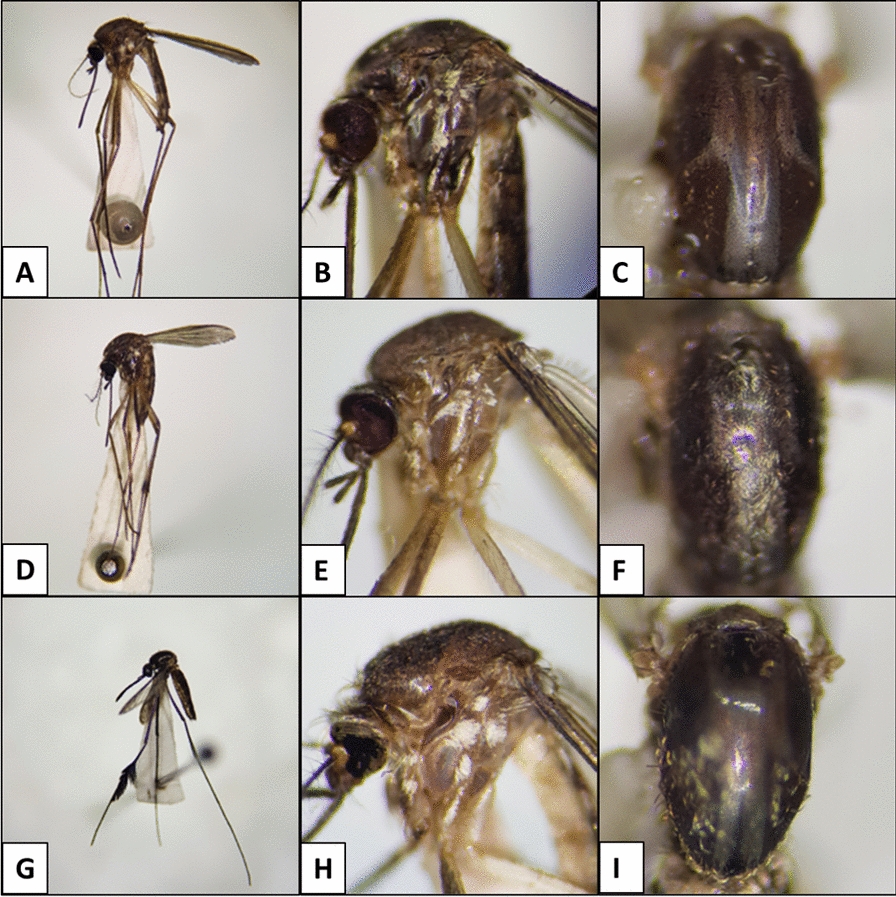
Examples of incorrectly identified images, due to damaged specimens. **A**–**C** and **H** *Ae. serratus* was mistaken for *Ae. scapularis*. **D** and **E** *Ae. scapularis* was mistaken for *Ae. serratus*. **F** *Ae. serratus* was mistaken for *Sa. albiprivus*. **G** *Sa. albiprivus* was mistaken for *Hg. leucocelaenus*. **I** *Ae. scapularis* was erroneously identified as *Hg. leucocelaenus*

We then evaluated the algorithm’s performance for each species based in the three groups of pictures. The algorithm’s best performance was observed when pictures from the three groups were combined in the same experiment. The performance of the AlexNet algorithm in identifying mosquitoes of the genus *Aedes* was poor, with the highest confidence intervals observed for the two species in this genus, compared with the others (95% CI 72–98) (Fig. 4). The algorithm misidentified at least one picture of *Ae. scapularis* as *Ae. serratus* or vice versa when presented with pictures of all three groups. In the run where all 1343 pictures were available, *Ae. serratus* was misidentified as *Hg. leucocelaenus*. In the run with pictures of the lateral view, only mosquitoes of the genus *Aedes* were misidentified, with the two *Aedes* species being swapped. In the run with the pronotum, several misidentifications occurred. *Ae. serratus* was incorrectly identified as either *Sa. albiprivus* or *Hg. leucocelaenus*, while *Ae. scapularis* was identified as *Sa. albiprivus* (Fig. 2). It is worth noting that many of the photographed specimens were not well-preserved, which may have contributed to the high rate of misidentification (Fig. 5).

## Discussion

In this study, we aim to identify four mosquito species that transmit yellow fever or other arboviruses by using a CNN (AlexNet). We also wanted to investigate whether the algorithm performance in classifying the mosquitoes changes according to the body regions shown on the pictures submitted. Our study found that the AlexNet can accurately identify mosquito pictures of the genus *Aedes*, *Sabethes* and *Haemagogus* with over 90% accuracy. Furthermore, the algorithm performance did not change according to the body regions shown.

Lorenz et al. [27] classified mosquitoes based on morphometric characteristics of their wings using neural networks, achieving accuracies ranging from 86% to 100%. However, an identification system based only on a body structure such as the wing is more fragile because if the structure is not present in the analysed photo, the identification is compromised. Therefore, a good identification system should work with any part of the insect’s body. Sauer et al. [28] showed that best-performing CNN configuration achieved a precision of 99% to discriminate between *Aedes* and non-*Aedes* mosquito species; the mean precision to predict the *Aedes* species was 91% for RGB pictures. Motta et al. [16] used three pre-trained networks to identify urban mosquitoes (*Aedes* and *Culex*), achieving an accuracy of 76.2% for the GoogleNet, 52.4% for LeNet and 51.2% for AlexNet. Okayasu et al. [29] showed better accuracy results (92.3%) with the identification of *Ae. albopictus*, *Anopheles stefensi* and *Cx. pipiens pallens* using AlexNet based on data augmentation and 12,000 training pictures. More recently, Motta et al. [17] optimized the CNN hyperparameters and obtained 97.3% accuracy in distinguishing between the mosquitoes of the genus *Aedes* and the *Culex* mosquitoes. Similarly, Goodwin et al. [30] and Park et al. [13] achieved 97% accuracy rates for mosquito identification (*Anopheles*, *Culex*, *Psorophora* and *Aedes* species) using deep learning neural networks. These networks rely on morphological features like those used by taxonomists [13]. Kittichai et al. [15] using two YOLO v3 model identified *Ae. aegypti*, *Ae. albopictus* and *Cx. quinquefasciatus* at a mean average accuracy of 98–100%. A recent study has shown that the accuracy and robustness of the CNN may reach 99% accuracy by incorporating spatial dropout layers to regularize the network and by modifying its structure to incorporate multi-view inputs [31]. Concatenating two YOLO v3 model exhibited the optimal performance in identifying mosquitoes, with mean average accuracy of 99%. The Swin MSI successfully identified 100% subspecies-level in *Culex pipiens* complex. Based on pictures of all body regions, AlexNet identified *Ae. scapularis*, *Ae. serratus*, *Hg. leucocelaenus* and *Sa.albiprivus* at 94% accuracy on average (Fig. 3). Compared with previous studies that have used neural networks for mosquito identification, our accuracy rate of 94% is aligned with most results obtained by others.

In our study, we did not find a significant difference in AlexNet performance in identifying mosquitoes based on different body regions. In fact, other studies have shown that CNNs are able to detect morphological differences in various body regions of *Aedes* mosquitoes [13], some of which are consistent with the most used dichotomous keys [32]. Such results reveal that deep learning models learn the distinctive morphological features of mosquitoes body areas; these are the same ones used by taxonomists. For instance, *Ae. scapularis* can be identified by its serrated abdomen, a proboscis that is larger than the anterior femur and the mesonotum with white scales forming a circle. *Ae. serratus* is identified by its serrated abdomen, a proboscis that is similar to or smaller than the anterior femur and a mesonotum that may or may not have a longitudinal stripe of white scales [7]. The two species are very similar because they belong to the subgenus *Ochlerotatus* (Lynch Arribálzaga, 1891), where most of the species in this group are indistinguishable based on morphological characters [33]. *Sa. albiprivus* has medium-sized legs with bluish scales, a golden-scaled abdomen that forms quadrilaterals and a proboscis that is much smaller than the anterior femur. *Sa. albiprivus* and *Hg. leucocelaenus* are two species with different morphological characteristics. *Sa. albiprivus* can be distinguished from *Hg. leucocelaenus* by its predominantly dull, dark colour and pleura with two vertical lines of silvery scales [7]. Other studies show that the accuracy of CNNs in identifying other insects is not significantly affected by the body region shown on the picture [22]. Our findings show that the morphological characteristics used for the identification of the mosquitoes included in this study are present in multiple regions of the body and, therefore, any of the body regions here studied allowed the AlexNet to accurately identify the mosquito species.

Deep learning neural networks consist of multiple convolutional layers, and databases with more pictures are more conducive to learning [21]. Additionally, many studies indicate that a larger picture bank improves the algorithm’s performance [13, 14, 16, 17, 22, 34, 35]. Even though a database with thousands of pictures is always desired, using a database with only 1343 pictures, we reached accuracy rates similar to those using databases 10× bigger than ours [17, 29]. AlexNet accuracy to identify *Sabethes* and *Haemogogus* mosquitoes was similar to the accuracy obtained with other CNNs used to identify other genera [17, 29]. However, the accuracy of the AlexNet in identifying *Ae. serratus* and *Ae. scapularis* was below 90% and, thus, suboptimal when compared with the performance of other CNNs (VGG-16, ResNet-50, SqueezeNet) that apply data augmentation and fine-tuning techniques to identify *Ae. aegypti* [16, 17], *Ae. albopictus* and *Ae. vexans* [13].

These accuracy values may be due to differences in algorithm architecture and training [13]. AlexNet is relatively shallow compared with deeper algorithms such as ResNet or DenseNet, which can capture complex hierarchical features in the pictures. Although AlexNet was designed to take advantage of GPU acceleration, its computational efficiency is suboptimal compared to other algorithms such as MobileNet. Finally, the features learned by AlexNet may not be as discriminative as those learned by algorithms trained on large datasets. Despite the limitations of AlexNet when compared with other algorithms, our results show high accuracy for identification of yellow fever mosquitoes. Our perspective is to apply other algorithms (ResNet, DenseNet or MobileNet) to test their efficiency in identifying *Ae. serratus*, *Ae. scapularis* and other sibling mosquito species. Moreover, optimization of CNN hyperparameters increase the accuracy of mosquito identification [17]. The lower performance of the algorithm, in some cases, may have been influenced by the state of preservation of the specimens. Analysis of the misidentified pictures in all experiments showed that the photographed specimens were not well preserved, especially in the pronotum area, where bristles and scales were missing, as well as the legs. Due to their size and the presence of scales and bristles, mosquitoes are easily damaged during capture, freezing and drying, resulting in the loss of critical morphological features necessary for proper identification. The state of preservation of the mosquitoes was a limiting factor in this work, and good preservation of specimens is important for optimal algorithm performance. Furthermore, the network has not been evaluated with images captured by different photographic devices. Therefore, is not possible to say whether images taken by cameras with different specifications influence AlexNet performance.

## Conclusions

We aim to identify four mosquito species that transmit yellow fever using AlexNet. We found that the AlexNet CNN can identify mosquito pictures of the genus *Aedes*, *Sabethes* and *Haemagogus* with over 90% accuracy, regardless of the body region being shown. Our results support the idea of applying CNNs for AI-driven identification of mosquito vectors of tropical diseases. This approach can potentially be used in the surveillance of yellow fever vectors by health services and the population as well.

### Supplementary Information

Additional file 1: Table S1. Accuracy observed for each experiment.

Additional file 2: Database S1.

## Data Availability

Data supporting the conclusions of this article are included in the article and its additional files.

## Abbreviations

CI: Confidence interval
CNN: Convolutional neural network
