# Supplementary material for: AI-driven convolutional neural networks for accurate identification of yellow fever vectors
Source: Parasit Vectors. 2024 Aug 2;17:329. doi: 10.1186/s13071-024-06406-2 (PMC11297716; doi:10.1186/s13071-024-06406-2)
Supplement: Supplementary file 1 — Additional file 1: Table S1. Accuracy observed for each experiment. [file 13071_2024_6406_MOESM1_ESM.docx]

| **Experiment** | **Species** | **(pseudo)-replicate** | **Accuracy** | | | | |
| --- | --- | --- | --- | --- | --- | --- | --- |
|  |  |  | "N" | Correct | Proportion | CI lower | CI upper |
| All pictures | *Aedes scapularis* | AN01 | 32 | 30 | 0.938 | 0.799 | 0.983 |
|  |  | AN02 | 32 | 23 | 0.719 | 0.546 | 0.844 |
|  |  | AN03 | 32 | 30 | 0.938 | 0.799 | 0.983 |
|  |  | AN04 | 32 | 27 | 0.844 | 0.682 | 0.931 |
|  |  | AN05 | 32 | 30 | 0.938 | 0.799 | 0.983 |
|  |  | AN06 | 32 | 29 | 0.906 | 0.758 | 0.968 |
|  |  | AN07 | 32 | 27 | 0.844 | 0.682 | 0.931 |
|  |  | AN08 | 32 | 28 | 0.875 | 0.719 | 0.950 |
|  |  | AN09 | 32 | 26 | 0.813 | 0.647 | 0.911 |
|  |  | AN10 | 32 | 29 | 0.906 | 0.758 | 0.968 |
|  |  | Sub-total | 320 | 279 | 0.872 | 0.831 | 0.904 |
|  | *Aedes serratus* | AN01 | 50 | 45 | 0.900 | 0.786 | 0.957 |
|  |  | AN02 | 50 | 48 | 0.960 | 0.865 | 0.989 |
|  |  | AN03 | 50 | 43 | 0.860 | 0.738 | 0.930 |
|  |  | AN04 | 50 | 47 | 0.940 | 0.838 | 0.979 |
|  |  | AN05 | 50 | 41 | 0.820 | 0.692 | 0.902 |
|  |  | AN06 | 50 | 47 | 0.940 | 0.838 | 0.979 |
|  |  | AN07 | 50 | 50 | 1.000 | 0.929 | 1.000 |
|  |  | AN08 | 50 | 47 | 0.940 | 0.838 | 0.979 |
|  |  | AN09 | 50 | 45 | 0.900 | 0.786 | 0.957 |
|  |  | AN10 | 50 | 49 | 0.980 | 0.895 | 0.996 |
|  |  | Sub-total | 500 | 462 | 0.924 | 0.897 | 0.944 |
|  | *Haemagogus leucocelaenus* | AN01 | 31 | 31 | 1.000 | 0.890 | 1.000 |
|  |  | AN02 | 31 | 31 | 1.000 | 0.890 | 1.000 |
|  |  | AN03 | 31 | 31 | 1.000 | 0.890 | 1.000 |
|  |  | AN04 | 31 | 31 | 1.000 | 0.890 | 1.000 |
|  |  | AN05 | 31 | 31 | 1.000 | 0.890 | 1.000 |
|  |  | AN06 | 31 | 31 | 1.000 | 0.890 | 1.000 |
|  |  | AN07 | 31 | 31 | 1.000 | 0.890 | 1.000 |
|  |  | AN08 | 31 | 31 | 1.000 | 0.890 | 1.000 |
|  |  | AN09 | 31 | 31 | 1.000 | 0.890 | 1.000 |
|  |  | AN10 | 31 | 31 | 1.000 | 0.890 | 1.000 |
|  |  | Sub-total | 310 | 310 | 1.000 | 0.988 | 1.000 |
|  | *Sabethes albiprivus* | AN01 | 23 | 23 | 1.000 | 0.857 | 1.000 |
|  |  | AN02 | 23 | 23 | 1.000 | 0.857 | 1.000 |
|  |  | AN03 | 23 | 23 | 1.000 | 0.857 | 1.000 |
|  |  | AN04 | 23 | 23 | 1.000 | 0.857 | 1.000 |
|  |  | AN05 | 23 | 23 | 1.000 | 0.857 | 1.000 |
|  |  | AN06 | 23 | 23 | 1.000 | 0.857 | 1.000 |
|  |  | AN07 | 23 | 23 | 1.000 | 0.857 | 1.000 |
|  |  | AN08 | 23 | 23 | 1.000 | 0.857 | 1.000 |
|  |  | AN09 | 23 | 23 | 1.000 | 0.857 | 1.000 |
|  |  | AN10 | 23 | 22 | 0.957 | 0.790 | 0.992 |
|  |  | Sub-total | 230 | 229 | 0.996 | 0.976 | 0.999 |
|  | Sub-total |  | 1360 | 1280 | 0.941 | 0.927 | 0.952 |
| Full-body | *Aedes scapularis* | AN01 | 11 | 11 | 1.000 | 0.741 | 1.000 |
|  |  | AN02 | 11 | 9 | 0.818 | 0.523 | 0.949 |
|  |  | AN03 | 11 | 11 | 1.000 | 0.741 | 1.000 |
|  |  | AN04 | 11 | 9 | 0.818 | 0.523 | 0.949 |
|  |  | AN05 | 11 | 10 | 0.909 | 0.623 | 0.984 |
|  |  | AN06 | 11 | 8 | 0.727 | 0.434 | 0.903 |
|  |  | AN07 | 11 | 9 | 0.818 | 0.523 | 0.949 |
|  |  | AN08 | 11 | 9 | 0.818 | 0.523 | 0.949 |
|  |  | AN09 | 11 | 10 | 0.909 | 0.623 | 0.984 |
|  |  | AN10 | 11 | 8 | 0.727 | 0.434 | 0.903 |
|  |  | Sub-total | 110 | 94 | 0.855 | 0.777 | 0.908 |
|  | *Aedes serratus* | AN01 | 210 | 184 | 0.876 | 0.825 | 0.914 |
|  |  | AN02 | 21 | 20 | 0.952 | 0.773 | 0.992 |
|  |  | AN03 | 21 | 18 | 0.857 | 0.654 | 0.950 |
|  |  | AN04 | 21 | 17 | 0.810 | 0.600 | 0.923 |
|  |  | AN05 | 21 | 18 | 0.857 | 0.654 | 0.950 |
|  |  | AN06 | 21 | 21 | 1.000 | 0.845 | 1.000 |
|  |  | AN07 | 21 | 17 | 0.810 | 0.600 | 0.923 |
|  |  | AN08 | 21 | 18 | 0.857 | 0.654 | 0.950 |
|  |  | AN09 | 21 | 19 | 0.905 | 0.711 | 0.973 |
|  |  | AN10 | 21 | 18 | 0.857 | 0.654 | 0.950 |
|  |  | Sub-total | 21 | 18 | 0.857 | 0.654 | 0.950 |
|  | *Haemagogus leucocelaenus* | AN01 | 15 | 15 | 1.000 | 0.796 | 1.000 |
|  |  | AN02 | 15 | 15 | 1.000 | 0.796 | 1.000 |
|  |  | AN03 | 15 | 15 | 1.000 | 0.796 | 1.000 |
|  |  | AN04 | 15 | 15 | 1.000 | 0.796 | 1.000 |
|  |  | AN05 | 15 | 15 | 1.000 | 0.796 | 1.000 |
|  |  | AN06 | 15 | 15 | 1.000 | 0.796 | 1.000 |
|  |  | AN07 | 15 | 15 | 1.000 | 0.796 | 1.000 |
|  |  | AN08 | 15 | 15 | 1.000 | 0.796 | 1.000 |
|  |  | AN09 | 15 | 15 | 1.000 | 0.796 | 1.000 |
|  |  | AN10 | 15 | 15 | 1.000 | 0.796 | 1.000 |
|  |  | Sub-total | 150 | 150 | 1.000 | 0.975 | 1.000 |
|  | *Sabethes albiprivus* | AN01 | 10 | 10 | 1.000 | 0.722 | 1.000 |
|  |  | AN02 | 10 | 10 | 1.000 | 0.722 | 1.000 |
|  |  | AN03 | 10 | 10 | 1.000 | 0.722 | 1.000 |
|  |  | AN04 | 10 | 10 | 1.000 | 0.722 | 1.000 |
|  |  | AN05 | 10 | 9 | 0.900 | 0.596 | 0.982 |
|  |  | AN06 | 10 | 10 | 1.000 | 0.722 | 1.000 |
|  |  | AN07 | 10 | 10 | 1.000 | 0.722 | 1.000 |
|  |  | AN08 | 10 | 10 | 1.000 | 0.722 | 1.000 |
|  |  | AN09 | 10 | 9 | 0.900 | 0.596 | 0.982 |
|  |  | AN10 | 10 | 10 | 1.000 | 0.722 | 1.000 |
|  |  | Sub-total | 100 | 98 | 0.980 | 0.930 | 0.994 |
|  | Sub-total |  | 570 | 526 | 0.923 | 0.898 | 0.942 |
| Lateral thorax | *Aedes scapularis* | AN01 | 5 | 4 | 0.800 | 0.376 | 0.964 |
|  |  | AN02 | 5 | 5 | 1.000 | 0.566 | 1.000 |
|  |  | AN03 | 5 | 5 | 1.000 | 0.566 | 1.000 |
|  |  | AN04 | 5 | 3 | 0.600 | 0.231 | 0.882 |
|  |  | AN05 | 5 | 3 | 0.600 | 0.231 | 0.882 |
|  |  | AN06 | 5 | 5 | 1.000 | 0.566 | 1.000 |
|  |  | AN07 | 5 | 5 | 1.000 | 0.566 | 1.000 |
|  |  | AN08 | 5 | 5 | 1.000 | 0.566 | 1.000 |
|  |  | AN09 | 5 | 5 | 1.000 | 0.566 | 1.000 |
|  |  | AN10 | 5 | 5 | 1.000 | 0.566 | 1.000 |
|  |  | Sub-total | 50 | 45 | 0.900 | 0.786 | 0.957 |
|  | *Aedes serratus* | AN01 | 12 | 11 | 0.917 | 0.646 | 0.985 |
|  |  | AN02 | 12 | 10 | 0.833 | 0.552 | 0.953 |
|  |  | AN03 | 12 | 11 | 0.917 | 0.646 | 0.985 |
|  |  | AN04 | 12 | 10 | 0.833 | 0.552 | 0.953 |
|  |  | AN05 | 12 | 12 | 1.000 | 0.758 | 1.000 |
|  |  | AN06 | 12 | 10 | 0.833 | 0.552 | 0.953 |
|  |  | AN07 | 12 | 11 | 0.917 | 0.646 | 0.985 |
|  |  | AN08 | 12 | 8 | 0.667 | 0.391 | 0.862 |
|  |  | AN09 | 12 | 11 | 0.917 | 0.646 | 0.985 |
|  |  | AN10 | 12 | 12 | 1.000 | 0.758 | 1.000 |
|  |  | Sub-total | 120 | 106 | 0.883 | 0.814 | 0.929 |
|  | *Haemagogus leucocelaenus* | AN01 | 7 | 7 | 1.000 | 0.646 | 1.000 |
|  |  | AN02 | 7 | 7 | 1.000 | 0.646 | 1.000 |
|  |  | AN03 | 7 | 7 | 1.000 | 0.646 | 1.000 |
|  |  | AN04 | 7 | 7 | 1.000 | 0.646 | 1.000 |
|  |  | AN05 | 7 | 7 | 1.000 | 0.646 | 1.000 |
|  |  | AN06 | 7 | 7 | 1.000 | 0.646 | 1.000 |
|  |  | AN07 | 7 | 7 | 1.000 | 0.646 | 1.000 |
|  |  | AN08 | 7 | 7 | 1.000 | 0.646 | 1.000 |
|  |  | AN09 | 7 | 7 | 1.000 | 0.646 | 1.000 |
|  |  | AN10 | 7 | 7 | 1.000 | 0.646 | 1.000 |
|  |  | Sub-total | 70 | 70 | 1.000 | 0.948 | 1.000 |
|  | *Sabethes albiprivus* | AN01 | 6 | 6 | 1.000 | 0.610 | 1.000 |
|  |  | AN02 | 6 | 6 | 1.000 | 0.610 | 1.000 |
|  |  | AN03 | 6 | 6 | 1.000 | 0.610 | 1.000 |
|  |  | AN04 | 6 | 6 | 1.000 | 0.610 | 1.000 |
|  |  | AN05 | 6 | 6 | 1.000 | 0.610 | 1.000 |
|  |  | AN06 | 6 | 6 | 1.000 | 0.610 | 1.000 |
|  |  | AN07 | 6 | 6 | 1.000 | 0.610 | 1.000 |
|  |  | AN08 | 6 | 6 | 1.000 | 0.610 | 1.000 |
|  |  | AN09 | 6 | 6 | 1.000 | 0.610 | 1.000 |
|  |  | AN10 | 6 | 6 | 1.000 | 0.610 | 1.000 |
|  |  | Sub-total | 60 | 60 | 1.000 | 0.940 | 1.000 |
|  | Sub-total |  | 300 | 281 | 0.937 | 0.903 | 0.959 |
| Pronotum | *Aedes scapularis* | AN01 | 15 | 13 | 0.867 | 0.621 | 0.963 |
|  |  | AN02 | 15 | 14 | 0.933 | 0.702 | 0.988 |
|  |  | AN03 | 15 | 15 | 1.000 | 0.796 | 1.000 |
|  |  | AN04 | 15 | 12 | 0.800 | 0.548 | 0.930 |
|  |  | AN05 | 15 | 10 | 0.667 | 0.417 | 0.848 |
|  |  | AN06 | 15 | 13 | 0.867 | 0.621 | 0.963 |
|  |  | AN07 | 15 | 13 | 0.867 | 0.621 | 0.963 |
|  |  | AN08 | 15 | 15 | 1.000 | 0.796 | 1.000 |
|  |  | AN09 | 15 | 14 | 0.933 | 0.702 | 0.988 |
|  |  | AN10 | 15 | 13 | 0.867 | 0.621 | 0.963 |
|  |  | Sub-total | 150 | 132 | 0.880 | 0.818 | 0.923 |
|  | *Aedes serratus* | AN01 | 17 | 16 | 0.941 | 0.730 | 0.990 |
|  |  | AN02 | 17 | 15 | 0.882 | 0.657 | 0.967 |
|  |  | AN03 | 17 | 15 | 0.882 | 0.657 | 0.967 |
|  |  | AN04 | 17 | 17 | 1.000 | 0.816 | 1.000 |
|  |  | AN05 | 17 | 16 | 0.941 | 0.730 | 0.990 |
|  |  | AN06 | 17 | 15 | 0.882 | 0.657 | 0.967 |
|  |  | AN07 | 17 | 16 | 0.941 | 0.730 | 0.990 |
|  |  | AN08 | 17 | 16 | 0.941 | 0.730 | 0.990 |
|  |  | AN09 | 17 | 15 | 0.882 | 0.657 | 0.967 |
|  |  | AN10 | 17 | 17 | 1.000 | 0.816 | 1.000 |
|  |  | Sub-total | 170 | 158 | 0.929 | 0.881 | 0.959 |
|  | *Haemagogus leucocelaenus* | AN01 | 9 | 9 | 1.000 | 0.701 | 1.000 |
|  |  | AN02 | 9 | 9 | 1.000 | 0.701 | 1.000 |
|  |  | AN03 | 9 | 9 | 1.000 | 0.701 | 1.000 |
|  |  | AN04 | 9 | 9 | 1.000 | 0.701 | 1.000 |
|  |  | AN05 | 9 | 9 | 1.000 | 0.701 | 1.000 |
|  |  | AN06 | 9 | 9 | 1.000 | 0.701 | 1.000 |
|  |  | AN07 | 9 | 9 | 1.000 | 0.701 | 1.000 |
|  |  | AN08 | 9 | 9 | 1.000 | 0.701 | 1.000 |
|  |  | AN09 | 9 | 9 | 1.000 | 0.701 | 1.000 |
|  |  | AN10 | 9 | 9 | 1.000 | 0.701 | 1.000 |
|  |  | Sub-total | 90 | 90 | 1.000 | 0.959 | 1.000 |
|  | *Sabethes albiprivus* | AN01 | 7 | 7 | 1.000 | 0.646 | 1.000 |
|  |  | AN02 | 7 | 7 | 1.000 | 0.646 | 1.000 |
|  |  | AN03 | 7 | 7 | 1.000 | 0.646 | 1.000 |
|  |  | AN04 | 7 | 7 | 1.000 | 0.646 | 1.000 |
|  |  | AN05 | 7 | 7 | 1.000 | 0.646 | 1.000 |
|  |  | AN06 | 7 | 7 | 1.000 | 0.646 | 1.000 |
|  |  | AN07 | 7 | 7 | 1.000 | 0.646 | 1.000 |
|  |  | AN08 | 7 | 7 | 1.000 | 0.646 | 1.000 |
|  |  | AN09 | 7 | 7 | 1.000 | 0.646 | 1.000 |
|  |  | AN10 | 7 | 7 | 1.000 | 0.646 | 1.000 |
|  |  | Sub-total | 70 | 70 | 1.000 | 0.948 | 1.000 |
|  | Sub-total |  | 480 | 450 | 0.938 | 0.912 | 0.956 |
| Grand-total |  |  | 2710 | 2537 | 0.936 | 0.926 | 0.945 |
